# Anti-Gout Effects of the Medicinal Fungus *Phellinus igniarius* in Hyperuricaemia and Acute Gouty Arthritis Rat Models

**DOI:** 10.3389/fphar.2021.801910

**Published:** 2022-01-11

**Authors:** Hongxing Li, Xinyue Zhang, Lili Gu, Qín Li, Yue Ju, Xuebin Zhou, Min Hu, Qīn Li

**Affiliations:** ^1^ School of Pharmacy, Hangzhou Medical College, Hangzhou, China; ^2^ Key Laboratory of Neuropsychiatric Drug Research of Zhejiang Province, Hangzhou Medical College, Hangzhou, China

**Keywords:** *Phellinus igniarius*, hyperuricaemia, monosodium urate, gouty arthritis, metabolomics

## Abstract

**Background:** *Phellinus igniarius* (*P. igniarius*) is an important medicinal and edible fungus in China and other Southeast Asian countries and has diverse biological activities. This study was performed to comparatively investigate the therapeutic effects of wild and cultivated *P. igniarius* on hyperuricaemia and gouty arthritis in rat models.

**Methods:** UPLC-ESI-qTOF-MS was used to identify the chemical constituents of polyphenols from wild *P. igniarius* (WPP) and cultivated *P. igniarius* (CPP). Furthermore, WPP and CPP were evaluated in an improved hyperuricaemia rat model induced by yeast extract, adenine and potassium oxonate, which was used to examine xanthine oxidase (XO) activity inhibition and anti-hyperuricemia activity. WPP and CPP therapies for acute gouty arthritis were also investigated in a monosodium urate (MSU)-induced ankle swelling model. UHPLC-QE-MS was used to explore the underlying metabolic mechanisms of *P. igniarius* in the treatment of gout.

**Results:** The main active components of WPP and CPP included protocatechuic aldehyde, hispidin, davallialactone, phelligridimer A, hypholomine B and inoscavin A as identified by UPLC-ESI-qTOF-MS. Wild *P. igniarius* and cultivated *P. igniarius* showed similar activities in reducing uric acid levels through inhibiting XO activity and down-regulating the levels of UA, Cr and UN, and they had anti-inflammatory activities through down-regulating the secretions of ICAM-1, IL-1β and IL-6 in the hyperuricaemia rat model. The pathological progression of kidney damage was also reversed. The polyphenols from wild and cultivated *P. igniarius* also showed significant anti-inflammatory activity by suppressing the expression of ICAM-1, IL-1β and IL-6 and by reducing the ankle joint swelling degree in an MSU-induced acute gouty arthritis rat model. The results of metabolic pathway enrichment indicated that the anti-hyperuricemia effect of WPP was mainly related to the metabolic pathways of valine, leucine and isoleucine biosynthesis and histidine metabolism. Additionally, the anti-hyperuricemia effect of CPP was mainly related to nicotinate and nicotinamide metabolism and beta-alanine metabolism.

**Conclusions:** Wild *P. igniarius* and cultivated *P. igniarius* both significantly affected the treatment of hyperuricaemia and acute gouty arthritis models *in vivo* and therefore may be used as potential active agents for the treatment of hyperuricaemia and acute gouty arthritis.

## Introduction

Gout is a long-term and recurrent metabolic disease characterized by purine metabolism disorders and/or uric acid metabolism imbalances, and it can be caused by genetic factors that make individuals more susceptible (genetics-environment interactions) or by unhealthy diets (Pascual et al., 2015; Robinson 2018; Punzi et al., 2019; Zhu et al., 2021). The pathogenesis of hyperuricaemia is further promoted by the excessive consumption of purines and increased synthesis or decreased excretion of uric acids. Under increased blood uric acid levels in hyperuricaemia, monosodium urate (MSU) crystal/tophi deposits within intra- and/or peri-articular areas further induce excruciating pain and chronic inflammatory responses that may lead to joint structure damage, named gouty arthritis or gout (Perez-Ruiz et al., 2015; Dhanasekar and Rasool 2016). Gout occurs in approximately 1–4% of the general population, and the prevalence may even be up to 10% in a few countries (Ragab et al., 2017; Elfishawi et al., 2018; Pascart and Lioté 2019). Furthermore, the global incidences of gout are increasing due to poor dietary habits, insufficient exercises and metabolic syndrome, which will likely lead to increasing medical treatment costs (Singh 2013; Dehlin et al., 2020).

Some effective gout therapy treatments based on the pathogenesis of gout include inflammation reduction, pain relief, and improved joint function achieved by reducing the levels of UA in serum/urine and dissolving MSU crystals (Martinon 2010; Kapoor et al., 2011; Sokolove and Lepus 2013; Hainer et al., 2014). Commonly prescribed hyperuricaemia medications, such as allopurinol, febuxostat, probenecid and benzbromarone, aim at reducing uric acid production or increasing uric acid excretion, and prescribed gouty arthritis medications, such as colchicine, nonsteroidal anti-inflammatory drugs (NSAIDs), corticosteroids and analgesic drugs, aim at suppressing joint inflammation progression and relieving pain (Fam 2001; Niel and Scherrmann 2006; Aran et al., 2011; Neogi 2011; Becker et al., 2015). However, some adverse effects of the agents above have been reported, such as skin rash, hypersensitivity, gastrointestinal bleeding, gastrointestinal toxicity (nausea, vomiting, diarrhea), renal/hepatic toxicity, anemia, coma and even death (Ben-Chetrit and Levy 1998; Rostom et al., 2002; Terkeltaub 2009; Le Graverand-Gastineau 2010; Crofford 2013). Therefore, it is thus necessary to focus our research on the discovery of new alternative agents, such as natural herbal medicinal sources, with fewer or reduced side effects and greater efficacy and safety for the prevention and treatment of gout.

*Phellinus igniarius* (DC. Ex Fr.) Quel (*P. igniarius*), which belongs to the Polyporaceae family, is a perennial medicinal and edible fungus (Wu et al., 2019) that prefers to hosts the stems of aspen, robur, and birch in the wild (Mo et al., 2004; Wu et al., 2010). *P. igniarius*, which is referred to as “Sanghuang” in China, is widely consumed as a health care product for adjunct therapies or as preventive measures (Zhen-ting and Hai-ying 2016). *P. igniarius* has been used for the treatment of bellyache, fester and bloody gonorrhea in traditional Chinese medicine for thousands of years (Wang et al., 2005b). In several folk herbal medicine formulas or recipes, *P. igniarius* is used to treat stomach aches and arthritis (Zheng et al., 2018). Bioactive components from *P. igniarius*, such as flavones, polyphenols, polysaccharides or different extracts, have been reported to possess antiviral (Lee et al., 2013), anti-inflammatory (Sun et al., 2018), antioxidative (Wang et al., 2005b; Zhang et al., 2014), antitumor (Hsin et al., 2017), and immunomodulatory activities (Gao et al., 2017).

Considering the protective effects of *P. igniarius* and its anti-inflammatory and antioxidative activities, this study was designed to investigate the anti-hyperuricaemia and anti-gouty arthritis effects of *P. igniarius* in a rat model. In addition, the development and utilization of *P. igniarius* as a medicinal fungal resource has been restricted because its high medicinal value and the insufficient supply of wild-sourced *P. igniarius* result in it being very expensive. Fortunately, artificial cultivation is a practical way to efficiently obtain *P. igniarius* fruit. However, comparative studies of wild and cultivated *P. igniarius* have not been reported. One of the other goals of this study was to comparatively evaluate the efficacy of wild *P. igniarius* and cultivated *P. igniarius* in the treatment of hyperuricaemia and gouty arthritis.

In this study, we systematically characterized the chemical components of polyphenols from wild and cultivated *P. igniarius* and examined the uric acid-lowering effects in a hyperuricaemia rat model and the anti-inflammatory effects in an acute gouty arthritis rat model. The underlying metabolic mechanism of *P. igniarius* on gout was further investigated by UHPLC-QE-MS for urine metabolomics analysis.

## Materials and Methods

### Plant Material

Wild *P. igniarius* samples were purchased from Zhejiang Qiandao Lake Sangdu Edible Fungus Professional Cooperative (Hangzhou, China). The cultivated *P. igniarius* was an artificially cultivated strain from wild *P. igniarius* named “*Zhehuang No. 1*”, which was also provided by Zhejiang Qiandao Lake Sangdu Edible Fungus Professional Cooperative (Hangzhou, China) and was identified as *Phellinus igniarius* by the Horticulture Institute of Zhejiang Academy of Agricultural Sciences and Institute of Edible Fungi of Shanghai Academy of Agricultural Sciences. All samples were stored in a dark environment with a consistent temperature and humidity.

### Preparation of Polyphenols From *P. igniarius*

By referencing the polyphenol contents and following scientific principles and environmental protection protocols, response surface methodology was used to determine the optimal parameters for the extraction and purification of polyphenols from *P. igniarius*. Specifically, the dried fruit body of *P. igniarius* was crushed and passed through a 40-mesh screen. The powder was soaked in 70% ethanol for 30 min, extracted with 16-fold 70% ethanol for 2 h at 80°C in the dark and was filtered. The residue was re-extracted under the same conditions. These two filtrates were combined, and the solvent was removed under negative pressure at 60°C without light to yield the ethanol extract of wild *P. igniarius* and cultivated *P. igniarius*. Afterwards, the crude extract solution was adsorbed by the HP20 macroporous resin, impurities were removed with 10% ethanol at 5 times the solution volume and then eluted with 40% ethanol at 5 times the solution volume. The eluates were collected and pooled and then freeze-dried into a powder. The polyphenols from wild *P. igniarius* (WPP) and cultivated *P. igniarius* (CPP) were prepared sequentially, kept in the light and stored at −20°C. The Folin-Ciocalteu method is used to determine the concentration of total polyphenols.

### UPLC-ESI-qTOF-MS Analysis of the Constituents From Wild *P. igniarius* and Cultivated *P. igniarius*

WPP (10 mg) and CPP (10 mg) were dissolved in methanol (10 ml) under an ultrasound. The supernatant was separated by centrifugation at 10,000 rpm for 10 min and transferred into a sample bottle for testing.

UPLC-ESI-qTOF-MS analyses were performed using UPLC system (Waters) with an ACQUITY UPLC BEH C18 column (100 × 2.1 mm, 1.7 μm). The column temperature was 40°C, injection volume was 5 μL and flow rate was 0.35 ml/min. A 0.1% formic acid-water (v: v) solution was used as mobile phase A, and 0.1% formic acid-acetonitrile (v: v) was used as mobile phase B. The gradient elution conditions were set as follows: 0–2 min: 10% B; 2–26 min, 10%–90% B; 26–28 min, 90% B; 28–29 min, 90%–10% B; 29–30 min, 10% B.

A Waters Synapt G2 Q-TOF system (Waters Manchester, United Kingdom) equipped with an ESI ion source that operated in both the positive ion mode (ESI^+^) and negative ion mode (ESI^−^). The ESI source parameters were set as follows: a source temperature of 120°C, desolvation temperature of 350°C, and electrospray capillary voltage of 3.0 kV for the positive ionization mode and −2.5 kV for negative ionization mode. The cone voltage was set at 30 V. Argon and nitrogen were used as collision and cone gases, respectively. The cone and desolvation gas flows were 80 L/h and 800 L/h, respectively. A scan time of 0.2 s with an intrascan time of 0.1 s was utilized. The collision energy was set at 15–30 V, and the collision gas flow was set at 0.4. A lock-mass of leucine enkephalin was used for the positive ion mode ([M + H] ^+^ = 556.2771) and the negative ion mode ([M-H]^-^ = 554.2615) employed at a flow rate of 5 µL/min through a lockspray interface. The mass spectrometric data were collected from *m/z* 100 to 1,000 in the positive and negative ion modes under the centroid mode.

MassLynx V4.1 software (Waters Corporation) was used to process the data. Manual identification was performed to characterize the chemical constituents from WPP and CPP by comparing the exact mass and fragmentation pattern of the compounds that were previously reported in articles.

### Animals

Male SD rats (8 weeks, 180–220 g) were obtained from Zhejiang Laboratory Animal Center (licence No. SYXK 2016–0022). All rats were housed in clean plastic cages and maintained at 23 ± 1°C with 55% relative humidity, a 12-h light/dark cycle (7:00–19:00) and *ad libitum* access to pure water and standard rat pellets. The protocol of the study was reviewed and approved by the Animal Ethics Committees of Hangzhou Medical College. All experiments, including animal breeding, experimental operations, and animal euthanasia, were performed in accordance with the guidelines established by the committee.

### Establishment of a Rat Hyperuricaemia Model and Drug Administration

The experimental design was performed as described in previous studies with some modifications (Li et al., 2017). Fifty male SD rats were randomly divided into five groups (*n* = 10) and orally administered an equal 10 ml/kg dose of a normal saline solution (the control group and model group), 10 mg/kg allopurinol (Macklin Biochemical Technology Co., Ltd., China) (the positive control group), 150 mg/kg polyphenols from wild *P. igniarius* (the WPP-treated group) and 150 mg/kg polyphenols from cultivated *P. igniarius* (the CPP-treated group) for 7 days. Yeast extract (Beyotime Biochemical Technology Co., Ltd., China) (10 g/kg) and adenine (Macklin Biochemical Technology Co., Ltd., China) (100 mg/kg) were gavaged 12 h prior to allopurinol, WPP, and CPP administration every day except for the control group. The model group, positive control group, WPP-treated group and CPP-treated group were intraperitoneally injected with potassium oxonate (Yuanye Biochemical Technology Co., Ltd., China) (300 mg/kg) on days 1, 3, and 7, while the control group was injected with an equal volume of vehicle (0.5% CMC-Na).

### Establishment of a Rat Acute Gouty Arthritis Model and Drug Administration

Fifty male SD rats were randomly divided into five groups (*n* = 10): the control group, model group, positive control group, WPP-treated group and CPP-treated group. The animals in the WPP-treated group, CPP-treated group and positive control group were treated orally with WPP (150 mg/kg), CPP (150 mg/kg) or colchicine (Shanghai Aladdin Biochemical Technology Co., Ltd., China) (0.3 mg/kg) for 7 days. The animals in the control group and model group were treated orally with vehicle (a normal saline solution; 10 ml/kg). On the 8th day, an acute gouty arthritis model was induced by performing intra-articular injections of a MSU (Sigma-Aldrich, United States) suspension (25 mg/ml; 50 μL) into the right ankle of rats in the model group, positive control group, WPP-treated group and CPP-treated group under anesthesia. The contralateral bulge of the joint capsule was regarded as the criterion for successful injection. The MSU suspension in the control group was replaced by the vehicle (95% normal saline solution + 5% Tween 80; 50 μL) suspension. Establishment of the acute gouty arthritis model was roughly considered successful if there was obvious swelling 2 h after MSU injection.

### Biochemical Samples Collection and Analysis

Blood samples were collected from the femoral artery of anaesthetized rats by intraperitoneal injection of 10% chloral hydrate (0.3 ml/100 g) on the morning of the 8th day. All blood samples were placed at room temperature for 30 min, and the serum was separated by centrifugation at 3,000 rpm for 10 min at 4°C. All serum samples were stored at −80°C until analysis.

Urine samples were collected during a 12 h period from the evening of the 7th day using metabolic cages. The samples were then centrifuged (4°C, 1,000 rpm, 5 min) to remove particulate contaminants. The supernatants were transferred and stored at −80°C until analysis. The hair around the right ankle joint of the rat was shaved, and the ankle joint capsule was incised along the center of the ankle joint with a scalpel. A normal saline solution was used to repeatedly flush the joint cavity to collect ankle joint synovial fluid. The samples were centrifuged at 1,000 rpm for 20 min at 4°C. The supernatants were transferred and stored at −80°C until analysis.

Evaluations of the ICAM-1, IL-1β and IL-6 levels in the serum and synovial fluid were performed using commercial enzyme-linked immunosorbent assay kits (Enzyme-linked Biotechnology Co., Ltd., Shanghai, China). The levels of uric acid (UA), creatinine (Cr) and urea nitrogen (UN) in the serum and urine were determined by the colorimetric method using different commercial assay kits (Jiancheng Biotechnology Institute, Nanjing, China) according to the manufacturer’s instructions.

### Swelling Degree Measurement

Ankle oedema was evaluated as an increase in the injected ankle thickness in millimeters (mm) and measured using a digital caliper at the same position, which was marked with a black pen. Right ankle joint oedema was calculated as the difference between the basal value and the test value observed at different time points after the MSU suspension was injected (0, 2, 6, 12, 24, 36, 48 h). The whole measurement process was conducted by only one experimenter to ensure the measurements were accurate.

Swelling degree (%) = [thickness test value (mm)- thickness basal value (mm)]/thickness basal value (mm) × 100%.

### Evaluation of Xanthine Oxidase Activity *in vivo*

The liver tissue (100 mg) of each rat was mixed with cold normal saline solution (900 μL) to prepare a 10% liver tissue homogenate. The homogenate samples were centrifuged at 3,500 rpm for 10 min at 4°C. The supernatants were transferred and stored at −80°C until analysis. Part of the supernatant was mixed with 9 times the amount of normal saline solution to prepare a 1% liver tissue homogenate. The absorbance value of 10% liver tissue homogenate and the total protein content of 1% liver tissue homogenate were determined using commercial assay kits (Jiancheng Biotechnology Institute, Nanjing, China) according to the manufacturer’s instructions to calculate XO activity.

### Histopathological Studies

In the hyperuricaemia models, the kidneys were quickly removed from anaesthetized rats by chloral hydrate, fixed in a buffered 4% paraformaldehyde solution for 24 h, and embedded in paraffin. Finally, paraffin sections (5 μm) were cut and stained with hematoxylin and eosin (H&E).

### UHPLC-QE-MS for Urine Metabolomics Analysis

#### Metabolite Extraction

Each urine sample (1 ml) was added to 10 μL of NaN_3_ solution (0.5 mg/L) and quenched in liquid nitrogen for 10 s. Two hundred microliters of extract solution (acetonitrile: methanol = 1:1) containing an isotopically labelled internal standard mixture was added to 50 μL of sample. After vortexing for 30 s, the samples were sonicated for 10 min in an ice-water bath and incubated at −40°C for 1 h to precipitate the proteins. Then, the samples were centrifuged at 12,000 rpm for 15 min at 4°C. The supernatant was transferred to a fresh glass vial for analysis. The quality control (QC) samples were prepared by mixing all the samples in equal aliquots.

### UHPLC-QE-MS Analysis

The instrument settings for the UHPLC-QE-MS system were the same as previously described (He et al., 2020). The UHPLC separation process was performed using UHPLC system (Vanquish, Thermo Fisher Scientific) equipped with a UPLC BEH Amide column (2.1 × 100 mm, 1.7 μm) coupled to a Q Exactive HFX mass spectrometer (Orbitrap MS, Thermo). Ammonium acetate (25 mmol/L) and ammonia hydroxide (25 mmol/L) in water (pH = 9.75) were used for mobile phase A, and acetonitrile was used for mobile phase B. The injection volume was 3 μL, and the autosampler temperature was 4°C.

The QE HFX mass spectrometer was chosen for its ability to acquire MS/MS spectra in the information-dependent acquisition mode utilizing the acquisition software controls (Xcalibur, Thermo). In this mode, the acquisition software continuously evaluates the full scan MS spectrum. The electrospray ionization (ESI) source was set to the positive mode (ESI^+^) and negative mode (ESI^−^). The sheath gas flow rate and Aux gas flow rate were 30 Arb and 25 Arb, respectively. The capillary temperature was 350°C. The full MS resolution and MS/MS resolution were set to 60,000 and 7,500, respectively. The collision energy was set as 10/30/60 in the NCE mode. The spray voltage was set to 3.6 kV (positive) or −3.2 kV (negative).

### Data Annotation, Multivariate Statistical Analysis and Metabolic Pathway Analysis

The raw data, including information such as peak detection, extraction, alignment, and integration, were programmatically converted to the mzXML format and then ordered to perform metabolic annotations. The final datasets were imported into the SIMCA V15.0.2 software package (Sartorius Stedim Data Analytics AB, Umea, Sweden) for logarithmic conversion and centralized formatting processing. To visualize group separation and find significantly changed metabolites, principal component analysis (PCA) and orthogonal projections to latent structures-discriminate analysis (OPLS-DA) were carried out sequentially. The value of variable importance in the projection (VIP) was calculated in the OPLS-DA analysis.

Metabolites between the two groups with *p* < 0.05 (Student’s t-test) and VIP >1 were considered differential metabolites for subsequent analysis. The up- and down-regulation of differential metabolites were visualized by volcano plots. The differential metabolites obtained were subjected to pathway enrichment analysis in the KEGG database (http://www.genome.jp/kegg/) and MetaboAnalyst (http://www.metaboanalyst.ca/) to find the key metabolic pathways with the highest correlation, which further revealed the related metabolic mechanism of wild and cultivated *Phellinus igniarius* in the treatment of hyperuricaemia.

### Statistical Analysis

GraphPad Prism V8.3.0 software was used for the statistical analysis and data visualization. Multiple comparisons of data between groups were performed by one-way ANOVA or two-way ANOVA. The data are expressed as the mean ± standard deviation (SD), and *p* < 0.05 was considered statistically significant.

## Results

### Chemical Composition of Wild *P. igniarius* and Cultivated *P. igniarius*

**FIGURE 1:**
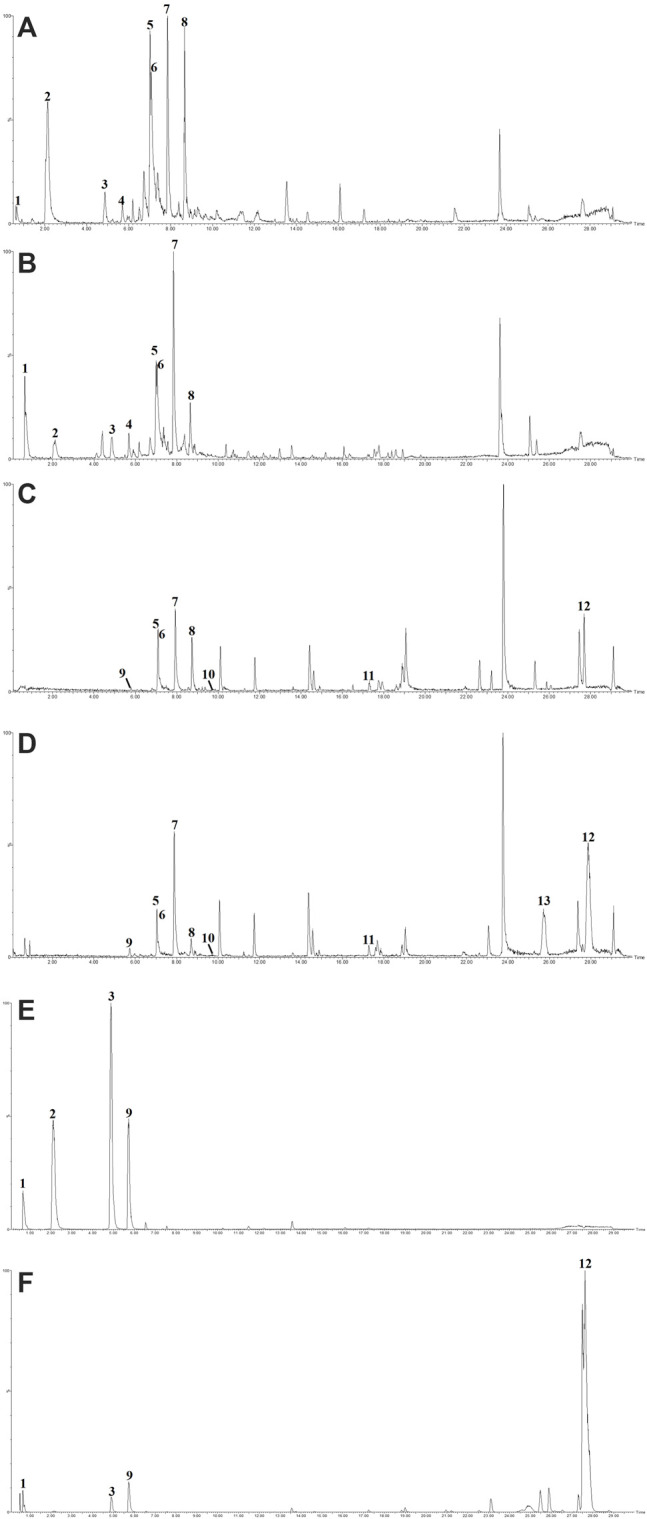
The BPIs of WPP by UPLC-ESI-qTOF-MS analysis in ESI^−^ **(A)** and ESI^+^ **(C)**, the BPIs of CPP by UPLC-ESI-qTOF-MS analysis in ESI^−^ **(B)** and ESI^+^ **(D)** and the BPIs of five mixed standards by UPLC-ESI-qTOF-MS analysis in ESI^−^ **(E)** and ESI^+^ **(F)**.

The concentration of total polyphenols in WPP is 498.21 ± 22.07 μg/mg (*n* = 3) and the concentration of total polyphenols in CPP is 346.36 ± 26.23 μg/mg (*n* = 3). The response values of different chemical compositions differ in the negative ion mode and positive ion mode. Based on the UPLC-ESI-qTOF-MS chromatogram of WPP and CPP, good separations were achieved within 30 min. By comparing the retention time (t_R_), exact mass and fragmentation patterns that were reported in the literature, 12 compounds were identified from WPP (Figures 1A, C), 13 compounds were identified from CPP (Figures 1B, D), and their results are shown in Tables 1–4. Among them, the compounds confirmed by comparison with the chemical reference standards are marked with the symbol “▲” in Tables 1–4, and the results are shown in Figures 1E, F. 12 compounds, including sucrose, protocatechuic aldehyde, osmundacetone, inotilone, davallialactone, phelligridimer A, hypholomine B, inoscavin A, hispidin, phelligridin I, phellinone, and erucic amide, were identified from both WPP and CPP, which showed that WPP and CPP have similar compound spectra. In addition, area normalization analysis at 280 nm was performed, and the peak area ratio of the main compounds identified in WPP (75.03%) and CPP (72.11%) unanimously exceeded 70%, indicating that the compounds identified were the main active ingredients of WPP and CPP.

**TABLE 1:** Characterization of the chemical constituents in WPP by UPLC-ESI-qTOF-MS (NEG).

No	t_R_ (min)	[M-H]^−^	[M-H]^−^	ppm	Identity	References
		Formula	*m/z*			
1^▲^	0.670	C_12_H_21_O_11_	341.1084	1.5	Sucrose	Zhao et al. (2018)
2^▲^	2.146	C_7_H_5_O_3_	137.0239	0	Protocatechuic aldehyde	Dong et al. (2015); Wang et al. (2021)
3^▲^	4.980	C_10_H_9_O_3_	177.0552	0.6	Osmundacetone	Dong et al. (2015); Wang et al. (2021)
4	5.908	C_12_H_9_O_4_	217.0501	2.8	Inotilone	Dong et al. (2015)
5	7.042	C_25_H_19_O_9_	463.1029	0.6	Davallialactone	Dong et al. (2015)
6	7.053	C_52_H_31_O_20_	975.1409	0.2	Phelligridimer A	Dong et al. (2015); Wang et al. (2005b)
7	7.875	C_26_H_17_O_10_	489.0822	−3.1	Hypholomine B	Dong et al. (2015); Wang et al. (2021)
8	8.741	C_25_H_17_O_9_	461.0873	−1.5	Inoscavin A	Dong et al. (2015); Wang et al. (2021); Zhao et al. (2018)

**TABLE 2:** Characterization of the chemical constituents in CPP by UPLC-ESI-qTOF-MS (NEG).

No	t_R_	[M-H]^−^	[M-H]^−^	ppm	Identity	References
	(min)	Formula	*m/z*			
1^▲^	0.757	C_12_H_21_O_11_	341.1084	0.6	Sucrose	Zhao et al. (2018)
2^▲^	2.146	C_7_H_5_O_3_	137.0239	−1.5	Protocatechuic aldehyde	Dong et al. (2015); Zhao et al. (2018)
3^▲^	4.859	C_10_H_9_O_3_	177.0552	−3.4	Osmundacetone	Dong et al. (2015); Wang et al. (2021)
4	5.928	C_12_H_9_O_4_	217.0501	−3.7	Inotilone	Dong et al. (2015)
5	7.008	C_25_H_19_O_9_	463.1029	4.5	Davallialactone	Dong et al. (2015)
6	7.136	C_52_H_31_O_20_	975.1409	1.3	Phelligridimer A	Wang et al. (2005b); Dong et al. (2015)
7	7.863	C_26_H_17_O_10_	489.0822	−1.6	Hypholomine B	Dong et al. (2015); Wang et al. (2021)
8	8.644	C_25_H_17_O_9_	461.0873	−1.3	Inoscavin A	Dong et al. (2015); Wang et al. (2021); Zhao et al. (2018)

**TABLE 3:** Characterization of the chemical constituents in WPP by UPLC-ESI-qTOF-MS (POS).

No	t_R_	[M + H] ^+^	[M + H] ^+^	ppm	Identity	References
	(min)	Formula	*m/z*			
9^▲^	5.787	C_13_H_11_O_5_	247.0606	−1.2	Hispidin	Dong et al. (2015)
5	7.096	C_25_H_21_O_9_	465.1186	1.3	Davallialactone	Dong et al. (2015)
6	7.172	C_52_H_33_O_20_	977.1565	0.5	Phelligridimer A	Dong et al. (2015); Wang et al. (2005b)
7	7.936	C_26_H_19_O_10_	491.0978	−0.2	Hypholomine B	Dong et al. (2015); Wang et al. (2021)
8	8.733	C_25_H_19_O_9_	463.1029	−4.1	Inoscavin A	Dong et al. (2015); Wang et al. (2021); Zhao et al. (2018)
10	9.777	C_33_H_21_O_13_	625.0982	−3.2	Phelligridin I	Wang et al. (2007)
11	17.312	C_15_H_23_O_2_	235.1698	−4.3	Phellinone	Zhao et al. (2018)
12	27.697	C_22_H_44_NO	338.3423	−0.6	Erucic amide	Zhao et al. (2018)

**TABLE 4:** Characterization of the chemical constituents in CPP by UPLC-ESI-qTOF-MS (POS).

No	t_R_	[M + H] ^+^	[M + H] ^+^	ppm	Identity	References
	(min)	Formula	*m/z*			
9^▲^	5.777	C_13_H_11_O_5_	247.0606	2.4	Hispidin	Dong et al. (2015)
5	7.074	C_25_H_21_O_9_	465.1186	0	Davallialactone	Dong et al. (2015)
6	7.117	C_52_H_33_O_20_	977.1565	−2.1	Phelligridimer A	Dong et al. (2015); Wang et al. (2005b)
7	7.883	C_26_H_19_O_10_	491.0978	−1	Hypholomine B	Dong et al. (2015); Wang et al. (2021)
8	8.702	C_25_H_19_O_9_	463.1029	−2.8	Inoscavin A	Dong et al. (2015); Wang et al. (2021); Zhao et al. (2018)
10	9.671	C_33_H_21_O_13_	625.0982	1.4	Phelligridin I	Wang et al. (2007)
11	17.275	C_15_H_23_O_2_	235.1698	−2.6	Phellinone	Zhao et al. (2018)
13	25.760	C_28_H_41_O	393.3157	−1.5	Ergosta-4,6,8,22-Tetraen-3-One	Zhao et al. (2018)
12^▲^	27.601	C_22_H_44_NO	338.3423	0.3	Erucic amide	Zhao et al. (2018)

### Effects of Wild *P. igniarius* and Cultivated *P. igniarius* on Hyperuricaemia

**FIGURE 2:**
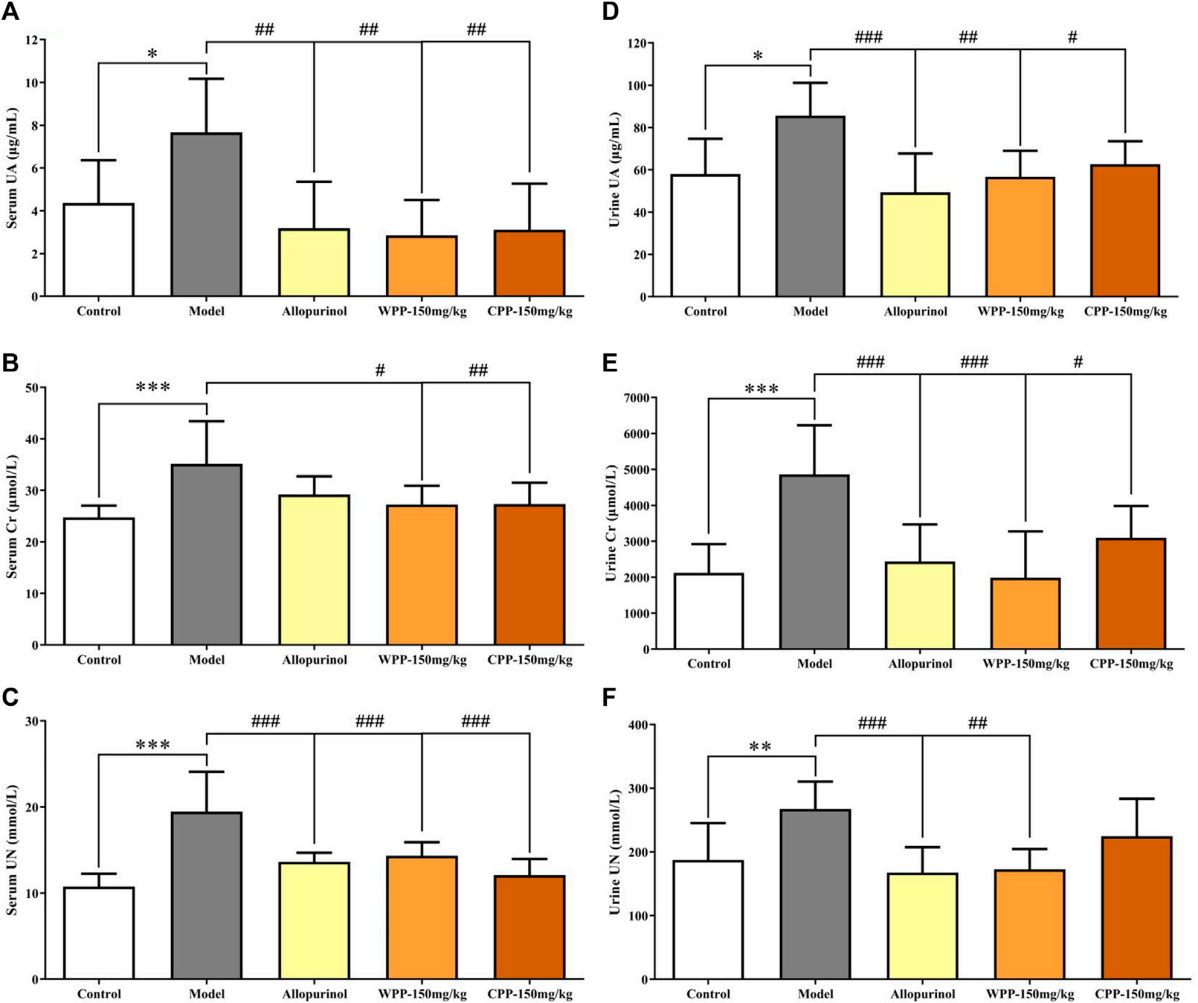
The effects of WPP and CPP on the index levels of renal functions in the serum and urine of hyperuricaemia rats. The levels of UA **(A)**, Cr **(B)** and UN **(C)** in serum and the levels of UA **(D)**, Cr **(E)** and UN **(F)** in urine are shown. Differences were analyzed by one-way ANOVA summary: *p* = 0.0032 **(A)**; *p* = 0.0015 **(B)**; *p* < 0.0001 **(C)**; *p =* 0.0023 **(D)**; *p* < 0.0001 **(E)**; *p* = 0.0012 **(F)**. Data are presented as the mean ± SD. *p < 0.05, **p < 0.01, ***p < 0.001 vs. control group; ^#^ *p* < 0.05, ^##^ *p* < 0.01, ^###^ *p* < 0.001 vs. model group; *n* = 10.

Uric acid, the culprit of hyperuricaemia, is used clinically to evaluate the quality of kidney function coupled with Cr and UN. As shown in Figure 2A, marked increase with significant difference (*p* < 0.05) in the levels of UA, Cr and UN was observed in the serum and urine of hyperuricaemia rats compared with that observed in the control group, indicating that the synthesis of uric acid was increased and the kidney damage was aggravated. Conversely, compared with the model group, the drug group treated orally with WPP (150 mg/kg) and CPP (150 mg/kg) showed significant decreases in the levels of UA, Cr and UN in the serum and urine (*p* < 0.05).

**FIGURE 3:**
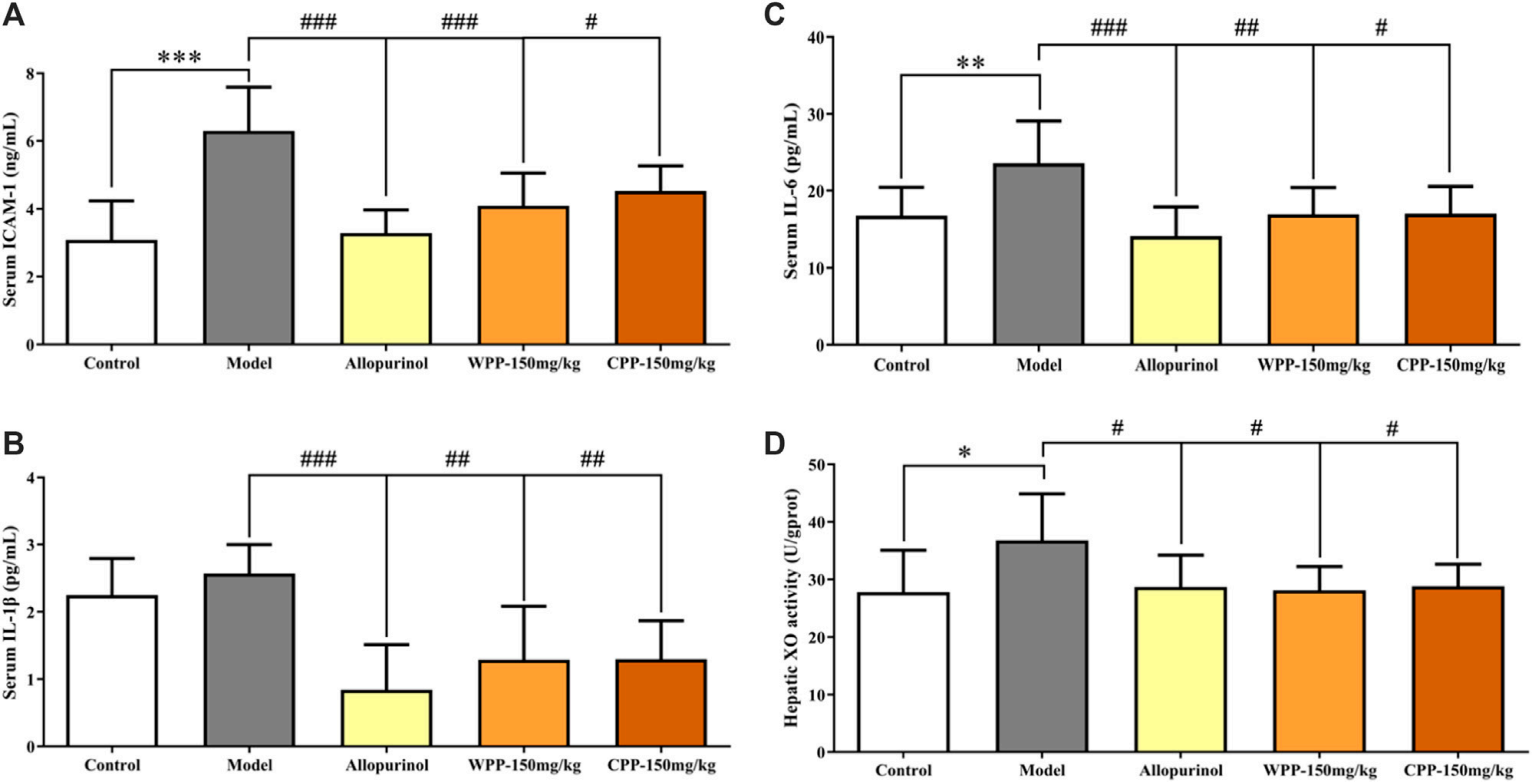
The effects of WPP and CPP on the expression levels of chemokines and pro-inflammatory cytokines in serum and hepatic XO activity of hyperuricaemia rats. The levels of ICAM-1 **(A)**, IL-1β **(B)** and IL-6 **(C)** in serum and hepatic XO activity **(D)** are shown, respectively. Differences were analyzed by one-way ANOVA summary: *p* < 0.0001 **(A)**; *p* = 0.0004 **(B)**; *p* = 0.0011 **(C)**; *p =* 0.0313 **(D)**. Data are presented as the mean ± SD. *p < 0.05, **p < 0.01, ***p < 0.001 vs. control group; ^#^ *p* < 0.05, ^##^ *p* < 0.01, ^###^ *p* < 0.001 vs. model group; *n* = 10.

The ICAM-1, IL-1β and IL-6 levels were measured to evaluate the anti-inflammatory effects of WPP and CPP on the treatment of hyperuricaemia. Figure 3 clearly shows that the combination of yeast extract, adenine and potassium oxonate in the model group significantly elevated the levels of ICAM-1 (*p* < 0.001), IL-1β and IL-6 (*p* < 0.01) in serum. Treatment with colchicine (0.3 mg/kg) significantly decreased (*p* < 0.001) the levels of ICAM-1, IL-1β and IL-6 simultaneously. WPP (150 mg/kg) and CPP (150 mg/kg) also significantly down-regulated the secretion of ICAM-1, IL-1β and IL-6 in serum with significant differences (*p* < 0.05) compared with that of the model group.

The activity of XO, one of the key enzymes that catalyse the production of uric acid from xanthine, was examined to evaluate the inhibition of uric acid production. Compared with the control group, the hepatic XO activity of hyperuricaemia rats was significantly increased (*p* < 0.05), indicating that the process of uric acid production was accelerated *in vivo*. After being treated with allopurinol (10 mg/kg), the hepatic XO activity in the positive control group was significantly down-regulated (*p* < 0.05), indicating that allopurinol can inhibit XO activity, which is consistent with its mechanism of action. Similarly, hepatic XO activity in the WPP-treated group and CPP-treated group was significantly down-regulated (*p* < 0.05) after oral administration of WPP (150 mg/kg) and CPP (150 mg/kg).

**FIGURE 4:**
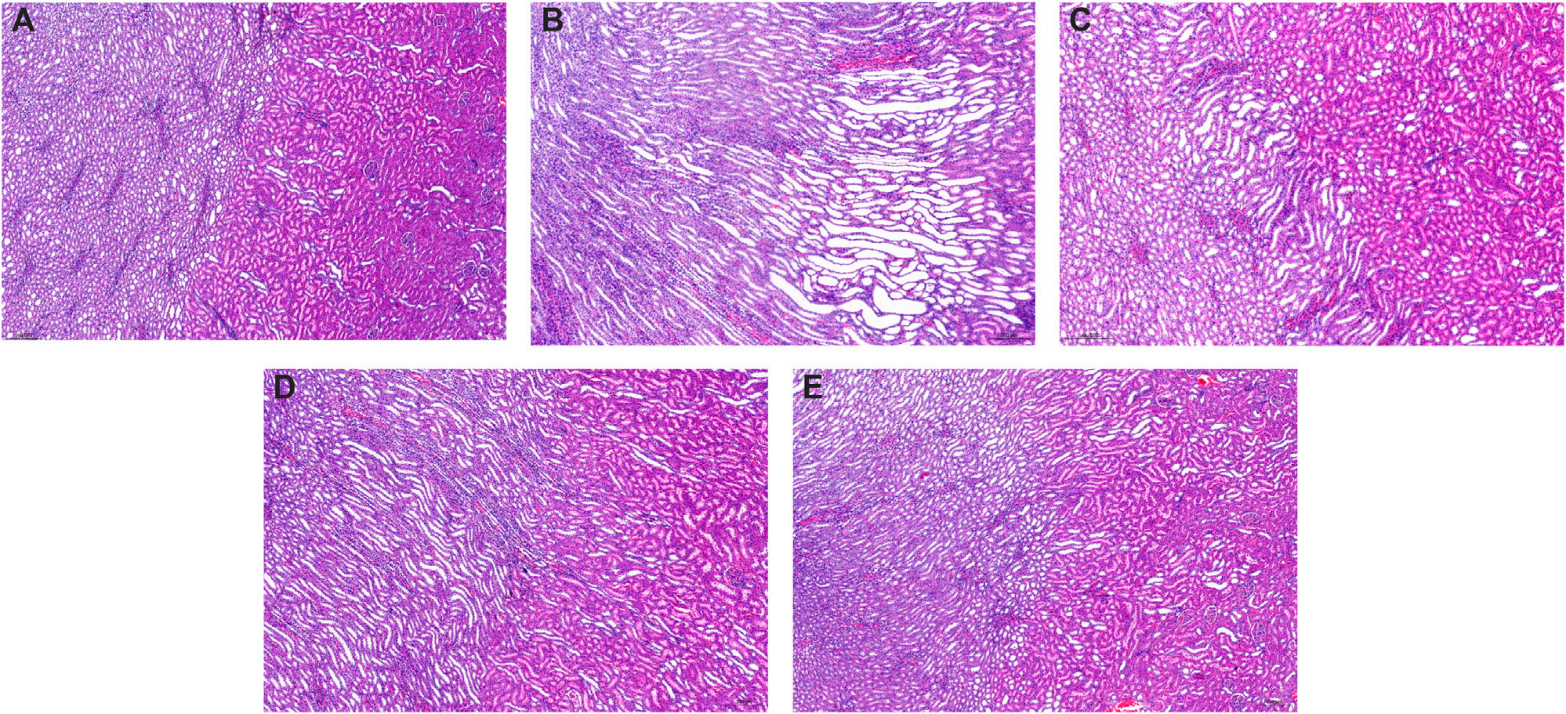
Representative kidney H&E images of the control group **(A)** and model group **(B)**, positive control group **(C)**, WPP-treated group **(D)** and CPP-treated group **(E)** are shown above. Scale bar = 200 μm.

Compared to the control group kidneys with normal renal structures (Figure 4A), the kidneys in model rats showed significant pathological changes including swelling, vacuolar degeneration of renal tubular epithelial cells, renal tubule atrophy, expansion of the lumen in the renal cortex, and atrophy and degeneration of the glomerulus (Figure 4B). After being treated with allopurinol (10 mg/kg), WPP (150 mg/kg) and CPP (150 mg/kg), the pathological damage was ameliorated to varying degrees (Figures 4C–E). The histological analysis data supported the UA level change observations and were consistent with the levels of Cr and UN observed above.

### UHPLC-QE-MS for Urine Metabolomics Analysis

**FIGURE 5:**
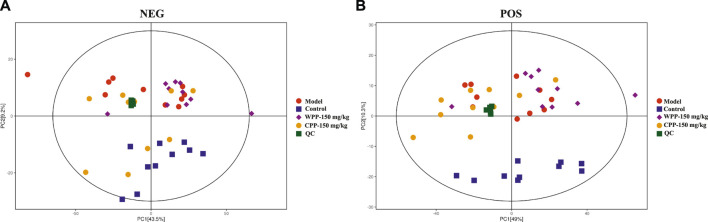
Principal component analysis (PCA) score plot of the control group, model group, WPP-treated group (150 mg/kg) and CPP-treated groups (150 mg/kg) in the negative ion mode (NEG) **(A)** and positive ion mode (POS) **(B)** (*n* = 10).

**FIGURE 6:**
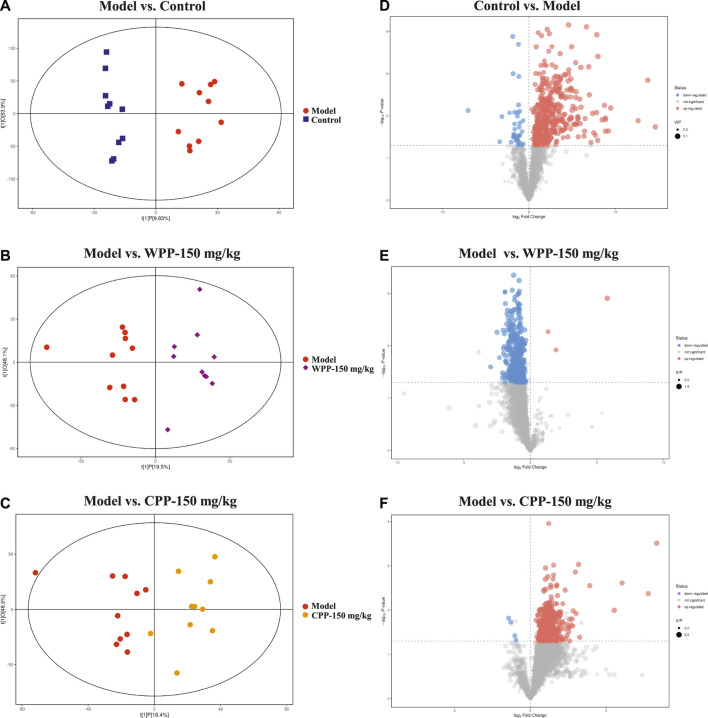
Orthogonal projections for the latent structures-discriminate analysis (OPLS-DA) score plot and volcano plot between the control group, model group, WPP-treated group (150 mg/kg) or CPP-treated groups (150 mg/kg) in the positive ion mode (POS) (*n* = 10). OPLS-DA score plots between the control group and model group **(A)**, model group and WPP-treated group (150 mg/kg) **(B)** and model group and CPP-treated group (150 mg/kg) **(C)**. The volcano plots between the control group and model group **(D)**, model group and WPP-treated group (150 mg/kg) **(E)** and model group and CPP-treated group (150 mg/kg) **(F)** are also shown above. The *X* axis and *Y* axis represent the log_2_-fold change and −log_10_ *p*-value, respectively.

Metabolite spectrum changes in gout treated with WPP (150 mg/kg) and CPP (150 mg/kg) were analyzed by PCA and compared with the control group and model group. The PCA score plot (Figure 5) showed that the metabolite spectra between the four groups were somewhat changed in both the ESI^+^ and ESI^−^. Furthermore, the OPLS-DA plot (Figures 6A–C) showed that the control group, model group, WPP-treated group and CPP-treated group could be clearly distinguished from each other. Differential metabolites between the two groups were screened out with *p* < 0.05 (Student’s t-test) and VIP >1 in ESI^+^ because the database records more information about substances in ESI^+^ than in ESI^−^. Specifically, there were 65 up-regulated metabolites and 8 down-regulated metabolites between the control and model groups (Figure 6D), 71 down-regulated metabolites between the model and WPP-treated groups (Figure 6E) and 50 up-regulated metabolites between the model and CPP-treated groups (Figure 6F).

**FIGURE 7:**
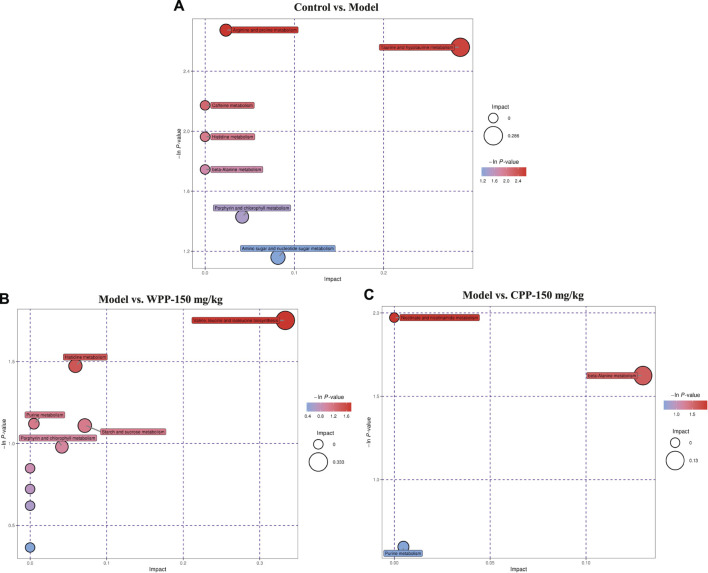
Metabolic pathway bubble plots of the control group and model group **(A)**, the model group and WPP-treated group (150 mg/kg) **(B)** and the model group and CPP-treated group (150 mg/kg) **(C)**. The *X* axis and *Y* axis represent the impact and −ln *p*-values, respectively.

Finally, based on the pathway impact value calculated from the pathway topological analysis and the -ln *p*-value obtained from pathway enrichment analysis, the top metabolic pathways were identified and are shown in the bubble plot above. The results (Figure 7) suggested that arginine and proline metabolism and taurine and hypotaurine metabolism may mediate the pathogenesis and development of hyperuricaemia. These changed metabolites were found to be primarily involved in valine, leucine and isoleucine biosynthesis metabolism and histidine metabolism of WPP and nicotinate and nicotinamide metabolism and beta-alanine metabolism of CPP for treatment effects on gout.

### Effects of Wild *P. igniarius* and Cultivated *P. igniarius* on Acute Gouty Arthritis

**FIGURE 8:**
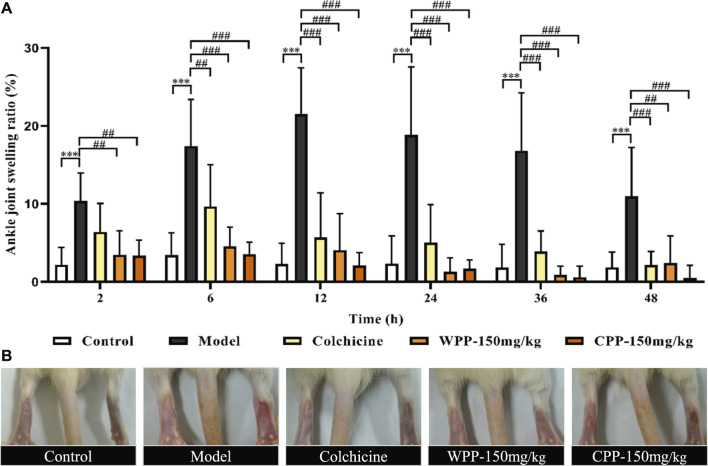
The effects of WPP and CPP on the degree of joint swelling in rats with MSU-induced acute gouty arthritis. **(A)** Measurement of the swelling degree of the right ankle joint of rats at different time points after MSU suspension injection (2, 6, 12, 24, 36, 48 h); **(B)** Representative images of the right ankle joint from each group at 12 h are shown. Differences were analyzed by two-way ANOVA interaction: *p* = 0.0027. Data are presented as the mean ± SD. *p < 0.05, **p < 0.01, ***p < 0.001 vs. control group at the corresponding hour; ^#^ *p* < 0.05, ^##^ *p* < 0.01, ^###^ *p* < 0.001 vs. model group at the corresponding hour; *n* = 10.

As shown in Figure 8A, after an equal volume of normal saline solution was injected, there was no obvious joint swelling in the control group rats at any time point, which indicated that the intra-articular injection had no effect on the degree of swelling. Compared with the control group, MSU significantly enhanced the swelling degree of the right ankle in the model group rats only 2 h after injection (*p* < 0.001). The joint swelling in the model group rats increased with time and reached a peak at 12 h (Figure 8B). After that, although the swelling degree of rats in the model group gradually decreased with increasing time, it was still significantly greater than that of the control group (*p* < 0.001) at different time points, which indicated that the acute gouty arthritis model was established successfully.

Colchicine (0.3 mg/kg) clearly interferes with MSU-induced joint swelling 2 h after injection. Specifically, the joint swelling in the positive group rats was significantly lower than that of the model control group at 6, 12, 24, 36, and 48 h (*p* < 0.01). Surprisingly, WPP (150 mg/kg) and CPP (150 mg/kg) showed good effects of suppressing the ankle swelling induced by MSU. Obviously, the joint swelling degree in the rats of the WPP-treated group and CPP-treated group at each time point was not observably changed compared with that of the control group, and the joint swelling degree was significantly lower than that of the model control group at the same time point with a significant difference (*p* < 0.01).

**FIGURE 9:**
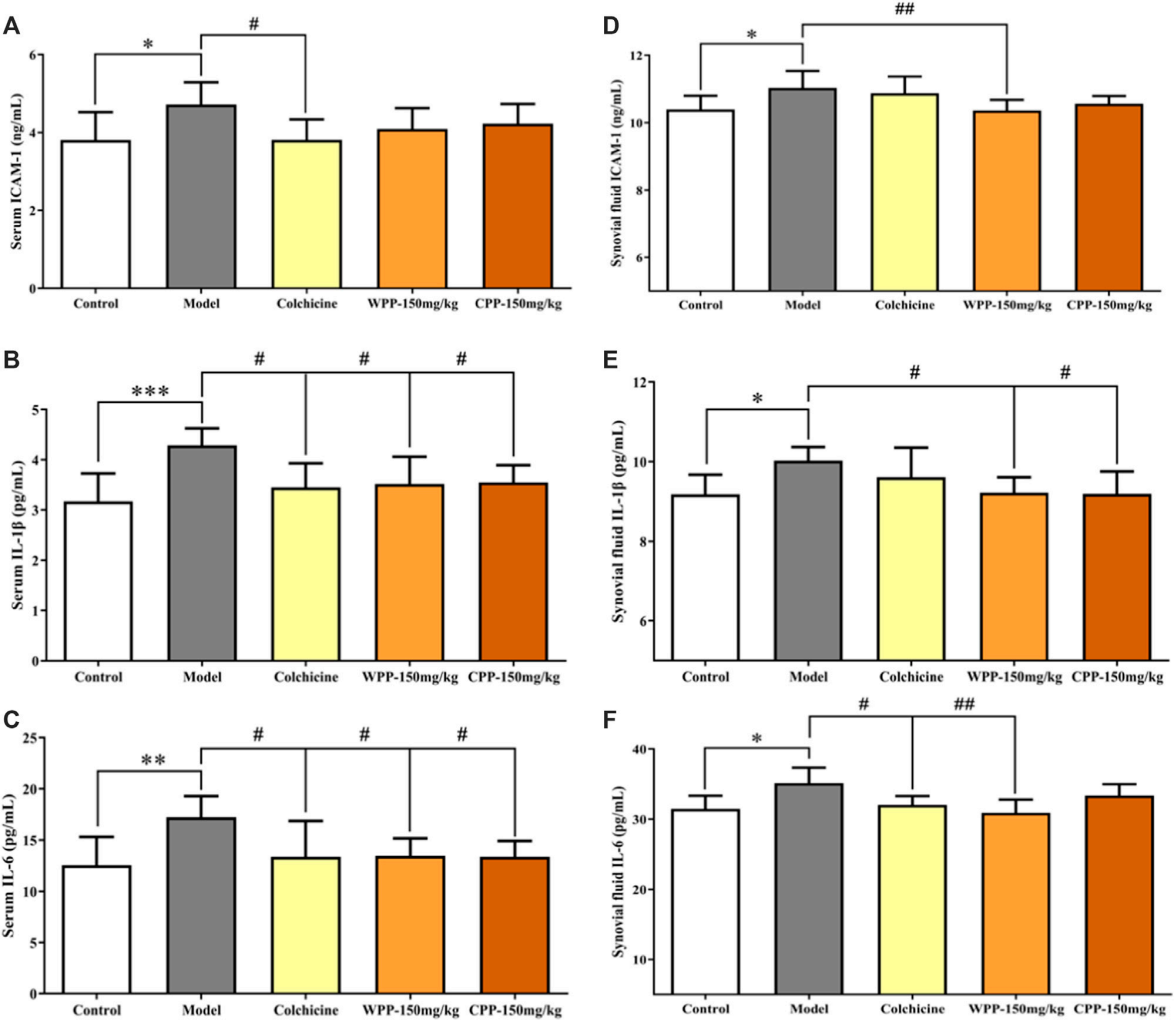
The effects of WPP and CPP on the expression levels of chemokines and pro-inflammatory cytokines in the serum and synovial fluid of MSU-induced acute gouty arthritis rats. The levels of ICAM-1 **(A)**, IL-1β **(B)** and IL-6 **(C)** in serum and the levels of ICAM-1 **(D)**, IL-1β **(E)** and IL-6 **(F)** in synovial fluid are shown. Differences were analyzed by one-way ANOVA summary: *p* = 0.0258 **(A)**; *p* = 0.0021 **(B)**; *p* = 0.0075 **(C)**; *p =* 0.0090 **(D)**; *p* = 0.0350 **(E)**; *p* = 0.0143 **(F)**. Data are presented as the mean ± SD. *p < 0.05, **p < 0.01, ***p < 0.001 vs. control group; ^#^ *p* < 0.05, ^##^ *p* < 0.01, ^###^ *p* < 0.001 vs. model group; *n* = 10.

The levels of three cytokines were examined to investigate the anti-inflammatory effects of WPP and CPP. The results (Figure 9) showed that MSU-induced acute gouty arthritis rats had significantly elevated levels of ICAM-1, IL-1β and IL-6 in serum (*p* < 0.05) and synovial fluid (*p* < 0.05). Treatment with WPP (150 mg/kg) and CPP (150 mg/kg) significantly down-regulated the production of ICAM-1, IL-1β and IL-6 in serum and synovial fluid compared with that in the model group. Colchicine (0.3 mg/kg) also decreased the levels of chemokines and pro-inflammatory cytokines.

## Discussion

Naturally sourced products have gradually become an indispensable source of novel pharmaceuticals, which rely on the presence of complex bioactive compounds (Shinkafi et al., 2015). In consideration of the precious medicinal and edible value and diverse biological activities of *P. igniarius*, a large number of studies on its anti-inflammatory, antioxidative, and immunomodulatory activities have been carried out, confirming its activities. Previous research has shown that *P. igniarius* extracts from liquid fermentation can affect the expression of xanthine oxidase (XO) and hypoxanthine-guanine phosphoribosyl transferase (HGPRT), two key enzymes in the uric acid metabolism pathway, to lower uric acid (Shuai-yang et al., 2019). One study also found that the ethanol extract of *P. igniarius* can inhibit XO activity and relieve acute gout inflammation by down-regulating the expression of IL-1β and ICAM-1 *in vitro* (Wang et al., 2021). Therefore, we focused our research on uncovering the active ingredients with anti-gout activity from *P. igniarius*.

In our prior research, a 70% ethanol extract of *P. igniarius* was screened out and was confirmed to possess significant anti-gout activity, which was determined by subjecting 13 extracts of *P. igniarius* to an enzymatic reaction and an *in vitro* cell model. *P. igniarius* is a rich source of secondary metabolites such as polyphenols, which are strong antioxidants (Wang et al., 2005a; Wang et al., 2005b; Lung et al., 2010; Lee and Yun 2011). Therefore, polyphenols from a 70% ethanol extract of wild *P. igniarius* (WPP) were prepared with HP20 macroporous resin to remove impurities and enrich the effective ingredients. In addition, few studies have focused on comparing the pharmacological activities of wild *P. igniarius* and cultivated *P. igniarius*. The polyphenols of cultivated *P. igniarius* (CPP) were prepared in the same way as above. Furthermore, to elucidate the main active components in WPP and CPP better, UPLC-ESI-qTOF-MS, which is characterized by fast separation, high sensitivity, and accurate relative molecular mass calculation (Ye et al., 2021), was used for identification. Surprisingly, similar active ingredients, such as protocatechuic aldehyde, hispidin, davallialactone, phelligridimer A, hypholomine B and inoscavin A, were identified in both WPP and CPP by UPLC-ESI-qTOF-MS. As members of the polyphenol family, the antioxidant and anti-inflammatory activities of protocatechuic aldehyde (Chang et al., 2011), hispidin (Jin et al., 2021), davallialactone (Lee et al., 2008; Lee et al., 2011), phelligridimer A (Wang et al., 2005b), hypholomine B (Shou et al., 2016) and inoscavin A (Lee et al., 2006; Shou et al., 2016) have been widely reported. A derivative of protocatechuic aldehyde effectively inhibited XO activity and reduced serum uric acid levels in hyperuricaemia mice (Lü et al., 2013). Hispidin also shows good inhibitory activity against bovine milk xanthine oxidase (BXO) *in vitro* and *in silico* (Linani et al., 2021). These active ingredients may provide an explanation for the various biological activities of *P. igniarius*.

Given that UA can be degraded to allantoin by the hepatic enzyme uricase in most mammals but not in humans (Shao et al., 2016), the hyperuricaemia rat model was established by using a uricase inhibitor (potassium oxonate) and large amounts of purine (yeast extract and adenine). In this study, we evaluated the therapeutic potential of WPP and CPP in the treatment of hyperuricaemia by measuring XO activity inhibition, the levels of UA, Cr and UN in serum and urine and the expression of ICAM-1, IL-1β and IL-6, and by observing kidney histological damage. XO, an important enzyme involved in the development of gout, can not only catalyse the hypoxanthine to xanthine and xanthine to uric acid reactions but can also directly catalyse the xanthine to uric acid reaction (Liu et al., 2012). We showed that hepatic XO activity in the drug group was significantly down-regulated after oral administration of WPP and CPP, which suggests that it is possible to screen potential XO inhibitors from *P. igniarius*. UA, the terminal product of purine metabolism, Cr and UN were confirmed to be correlated with the glomerular filtration rate and renal function (Kuroda et al., 2012). Compared with the model group, the expression levels of UA, Cr and UN in hyperuricaemia rats were significantly reduced after administering WPP and CPP, which can also be observed pathologically in the alleviation of kidney damage. The production of IL-1β, IL-6 and ICAM-1 was also decreased in the WPP-treated group and CPP-treated group to inhibit the inflammatory process in hyperuricaemia.

MSU deposition that is induced by increased blood uric acid levels in the joint cavity activates inflammatory cytokines, inducing the accumulation of monocytes-macrophages and neutrophils, which leads to gouty arthritis (Martinon et al., 2006; Li et al., 2019). During gouty arthritis attacks, MSU stimulates neutrophils and monocytes-macrophages to produce different proinflammatory cytokines, such as interleukin (IL)-1β and IL-6; this results in membranolysis and inflammasome activation, which further induce the expression of chemokines, such as intercellular adhesion molecule-1 (ICAM-1), that play a key role in transporting leukocytes across epithelial and endothelial barriers (Margalit et al., 1997; Neeson et al., 2003; Cianchetti et al., 2008; Shi et al., 2013). We further examined the anti-inflammatory effect of WPP and CPP on the production of proinflammatory cytokines in MSU-induced rats. These results clearly showed that WPP and CPP treatment both relieved joint swelling and inhibited the expression of IL-1β, IL-6 and ICAM-1, which are essential in the initiation and progression of acute gouty arthritis.

Metabolomics is an emerging field of research that has been used to evaluate metabolic perturbations associated with different stages of diseases, identify disease biomarkers, explore potential targets and predict drug safety and efficacy (Shan et al., 2020; Zhao et al., 2020; Song et al., 2021). Non-targeted metabolomics has been conducted extensively to explore biomarkers related to effective hyperuricaemia treatments (Qin et al., 2021; Zhang et al., 2021). Therefore, metabolomics based on UHPLC-QE-MS, which is suitable for simultaneous and systemic analysis of multiple metabolite fingerprinting, was employed to generate metabolite profiles of urine to characterize the metabolic changes related to hyperuricaemia and uncover the underlying metabolic mechanism of the therapeutic effects of WPP and CPP on gout. The results indicated that the progression of hyperuricaemia induced by potassium oxonate, yeast extract and adenine was mainly related to the metabolic pathways of arginine and proline metabolism and taurine and hypotaurine metabolism. The level of Cr in hyperuricaemia model rats was higher than that in normal rats, suggesting that arginine and proline metabolism was implicated in hyperuricaemia (Jiang et al., 2017; Shan et al., 2021). Up-regulated Cr in hyperuricaemia rats suggests that rats with hyperuricaemia have renal damage, which was consistent with previous studies (Liu et al., 2016). Higher plasma taurine levels have been observed in patients with uric acid stones (Atanassova et al., 2010). Taurine and hypotaurine metabolism has been confirmed to participate in several renal physiological processes and mediate renal uric acid excretion by regulating urate transporters (Feng et al., 2017). The potential therapeutic effect of WPP on gout was found to be primarily involved in leucine and isoleucine biosynthesis metabolism and histidine metabolism. The findings suggest that BCAA supplements (composed of leucine, isoleucine and valine) reduced the purine nucleotide cycle activity of the athletes, subsequently decreased uric acid production and the concentrations of hypoxanthine, and reduced the incidence of gout in individuals that engage in endurance exercises (Tang and Chan, 2017). The treatment effect of CPP on gout was primarily involved in nicotinate and nicotinamide metabolism and beta-alanine metabolism. Research has shown that quinolinic acid is involved in the metabolism of nicotinate and nicotinamide and is eventually metabolized to uric acid, resulting in increased uric acid (Zhao et al., 2020). Collectively, these findings suggest that WPP and CPP can exhibit an anti-gout protective effect by mediating different stages of uric acid metabolism.

## Conclusion

**FIGURE 10:**
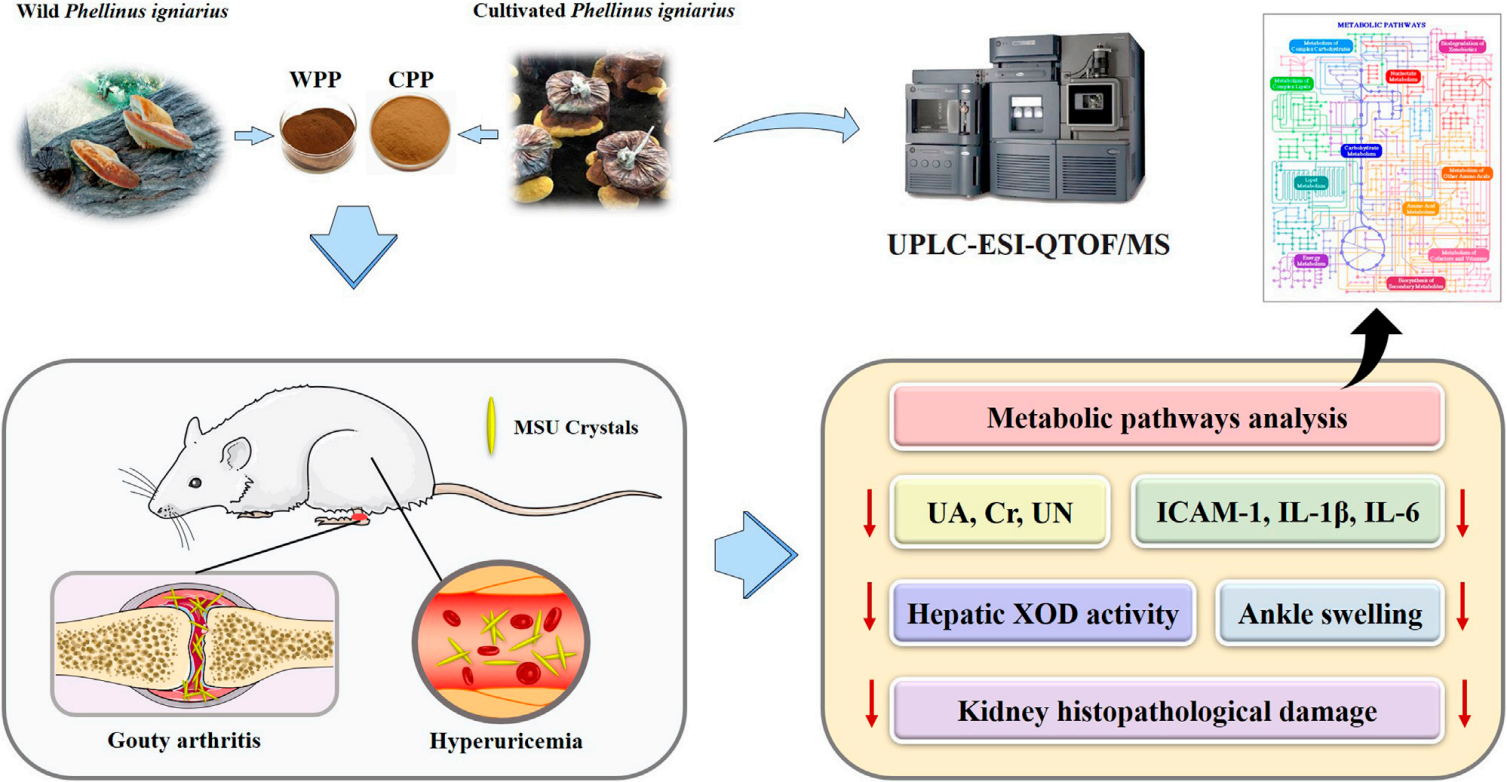
Overview of the anti-gout effects of wild *P. igniarius* and cultivated *P. igniarius*.

In summary, we provided evidence supporting that wild *P. igniarius* and cultivated *P. igniarius* have similar active ingredient spectrums and have anti-hyperuricaemia and anti-gout arthritis effects (Figure 10). The shared active compounds and analogous pharmacological activities are expected to promote the development and application of cultivated *P. igniarius* to fill the shortage of wild *P. igniarius*. In the future, we focused our research on uncovering the targets and pathways of *P. igniarius* in the treatment of gout.

## Data Availability

The original contributions presented in the study are included in the article/supplementary material, further inquiries can be directed to the corresponding author.

## Abbreviations

*P. igniarius*, *Phellinus igniarius*; WPP, polyphenols from wild *P. igniarius*; CPP, polyphenols from cultivated *P. igniarius*; XO, xanthine oxidase; MSU, monosodium urate; UA, uric acid; Cr, creatinine; UN, urea nitrogen; QC, quality control; ESI^+^, positive ion mode; ESI^-^, negative ion mode; PCA, principal component analysis; OPLS-DA, orthogonal projections to latent structures-discriminate analysis; VIP, value of variable importance in the projection.
